# Cross-Sectional and Longitudinal Relationships between Depressive Symptoms and Brain Atrophy in MS Patients

**DOI:** 10.3389/fnhum.2016.00622

**Published:** 2016-12-16

**Authors:** Heiner Stuke, Katrin Hanken, Jochen Hirsch, Jan Klein, Fabian Wittig, Andreas Kastrup, Helmut Hildebrandt

**Affiliations:** ^1^Department of Psychiatry and Psychotherapy, Charité Universitätsmedizin BerlinBerlin, Germany; ^2^Department of Psychology, University of OldenburgOldenburg, Germany; ^3^Department of Neurology, Klinikum Bremen-OstBremen, Germany; ^4^Fraunhofer MEVIS Institute for Medical Image ComputingBremen, Germany

**Keywords:** multiple sclerosis, depression, structural MRI, prediction, prospective study

## Abstract

**Introduction**: Depressive symptoms are a frequent and distressing phenomenon in Multiple Sclerosis (MS) patients. Cross-sectional research links these symptoms to reduced brain gray matter volumes in parts of the prefrontal and temporal lobe as well as subcortical structures like the hippocampus, nucleus caudatus and globus pallidus. Nevertheless, prospective relationships between regional gray matter volume and the course of depressive symptoms are poorly understood.

**Methods**: Forty-four patients with relapsing–remitting or secondary progressive MS participated in a prospective study with two assessments of depressive symptoms and high-resolution MRI with an inter-test-interval of 17 months. Relationships between baseline gray matter volume and baseline depressive symptoms, as well as prospective associations between the development of atrophy and depression were assessed using voxel-based morphometry (VBM).

**Results**: Cross-sectional analyses revealed an association between depressive symptoms and gray matter loss in the left temporal lobe. Prospective analysis showed that gray matter losses in the right middle cingulate and middle frontal gyrus at baseline predicted increasing depressive symptoms during follow-up. Increase in depressive symptoms was related to a concomitant increase in atrophy in the left thalamus and right globus pallidus.

**Discussion**: Our results fit well into the concept of a disturbed cortico–striatal–pallido–thalamic loop in depression. In this framework, progressive gray matter loss in limbic basal ganglia structures including globus pallidus and thalamus may lead to depression-typical deficits in hedonic motivation, whereas atrophy of the prefrontal cortex may contribute to maladaptive coping strategies, promoting an unfavorable development of depressive symptoms.

## Introduction

Depression is a frequent accompanying symptom of Multiple Sclerosis (MS) with a lifetime prevalence of 50% in patients, a replicated figure derived from tertiary care neurological clinics. This prevalence is three times higher than in the general population (Feinstein, 2007). Depression is one of the most important determinants of low quality of life in MS patients and seems to predict cognitive decline during the next years (Christodoulou et al., 2009). Accordingly, the relationship between structural brain changes and depressive symptoms in MS has been the focus of extensive empirical research. Here, brain atrophy or demyelination in the frontal lobe, the temporal lobe and the hippocampus has been related to increased depressive symptoms in MS patients (Gold et al., 2010; Kiy et al., 2011; Feinstein et al., 2014; Gobbi et al., 2014; Patejdl et al., 2016).

Both, depressive mood and structural brain abnormalities, are more common in MS patients compared to healthy subjects and thus it seems plausible that there is a MS-specific signature of depression-related brain structure changes. It is however a matter of ongoing debate whether the relationship between brain structure changes and depressive symptoms is comparable between samples with and without MS. To some degree, the results from research focusing on depressive patients without a somatic disease indeed mirror those from MS patients with depression. In these studies, reduced gray matter volumes in depressed patients without MS compared to healthy controls has been reported particularly for the frontal and anterior cingulate cortex (van Tol et al., 2010; Peng et al., 2011), the hippocampus (Campbell et al., 2004; Zou et al., 2010; Cole et al., 2011), the nucleus caudatus (Kim et al., 2008) and the globus pallidus (Lacerda et al., 2003; Bielau et al., 2005). For the amygdala, mixed results of decreased and increased sizes in depressed compared to healthy subjects has been reported, potentially due to varying treatment conditions of the investigated groups (Hamilton et al., 2008). A meta-analysis on 20 studies comparing regional brain volume of depressive patients with healthy control subjects found gray matter reductions in depressive patients in the bilateral anterior cingulate cortex, right middle and inferior frontal gyrus, right hippocampus and left thalamus (Du et al., 2012).

It is important to note, that all of these studies used a cross-sectional design, i.e., they have related brain atrophy to depression severity at one point in time. Thus, they could not elucidate associations between brain structure at one (baseline) time-point and future course of depressive symptoms or a concomitant development of depression and atrophy in time. The only prospective finding with respect to relationships between brain atrophy and depression is a small size of the hippocampus that has consistently been related to a poorer prospective clinical performance in depressive patients *without MS* (Frodl et al., 2008; Fu et al., 2013). Moreover, to the best of our knowledge, no study has hitherto directly related the course of depressive symptoms to the concomitant development of brain atrophy in the relevant brain regions (which requires MRI assessments at baseline as well as follow-up). With respect to this gap, the present study aims at:

1. Replicating previous findings on cross-sectional relationships between depression and brain atrophy patterns.
2. Informing whether experiencing depression at visit 1 might promote the development of a specific pattern of regional brain atrophy (for example in the hippocampi and therefore explain the depression-related loss of memory performance observed in two longitudinal studies (Christodoulou et al., 2009; Hildebrandt and Eling, 2014)).
3. Informing whether a specific pattern of brain atrophy at visit 1 is related to an increased risk for the patients to develop depressive mood in future.
4. Correlating the increase of brain atrophy over a period of time with the increase of depressive mood and therefore allowing for a more causal interpretation of the relation between dysfunctions of the brain and the experience of depression, which is not possible using cross-sectional studies.

Using a prospective study design with two assessments of depressive symptoms and structural MRI in MS patients with an interval of 17 months, we were able to test these associations between regional brain volume and depressive symptoms, as well as the change of brain volume and depressive symptoms between the two visits.

## Materials and Methods

### Subjects and Study Design

The initial sample comprised 46 MS patients that participated in an ongoing prospective, non-interventional study on MRI parameters characterizing progression of MS. The inclusion criteria were age of 18–65 years, diagnosis of relapsing–remitting (RRMS) or secondary progressive MS according to the McDonald criteria 2001 but no relapse during the last 4 weeks before the investigation, and an Expanded Disability Status Scale (EDSS) of 0–6.5. The ethical board of the Bremer Physicians Society approved the study and all patients gave informed consent. The study involved two visits with a mean interval of 17 months in between (the original study design laid down an interval of 18 months but due to the fast response of the patients at follow-up, we obtained a mean of 17 months). A different (fatigue-related) analysis of the same data set has already been published (Sander et al., 2016): we also refer to this article for further study details.

Of the initially 46 MS patients, two had to be excluded due to missing MRI or depression assessments at visit 1. Four additional subjects dropped out during follow-up, so that the final sample comprised 44 patients for cross-sectional analyses at visit 1 and 40 patients for prospective analyses.

### Assessment of Depressive Symptoms and MS-Related Clinical Status

Depressive symptoms were quantified at visit 1 and visit 2 using the Beck Depression Inventory (BDI; Beck et al., 1961). The course of depressive symptoms between the two visits was calculated by subtracting the BDI score at visit 1 from the BDI score at visit 2 (thus with negative values indicating an improvement and positive values indicating a worsening of depressive symptoms). The change in EDSS scores was calculated accordingly.

To detect possible differences between patients with RRMS and SPMS, we compared the two subtypes with regard to BDI scores at t1 and t2 as well as change in BDI scores between the two visits. To exclude the possibility, that changes in depression severity and its relationships with brain atrophy might be confounded by MS progress (e.g., due to the administration of corticosteroids that also affect brain volume), we computed correlations between change in BDI scores and change in EDSS scores as well as with the number of relapses during follow-up.

### MRI Image Acquisition and Preprocessing

High-resolution T1 MPRAGE MRI images were acquired at visit 1 and 2 using a 3T scanner (Siemens Skyra, Erlangen, Germany) at an isotropic resolution of 1 mm^3^ × 1 mm^3^ × 1 mm^3^. These images were normalized into MNI space and segmented into gray and white matter using the preprocessing procedure for longitudinal data as implemented in the voxel-based morphometry toolbox version 8 (VBM8) for statistical parametric mapping version 8 (SPM8). This approach uses the DARTEL (Ashburner, 2007) normalization algorithm and the standard SPM tissue probability map. Subsequently, maps showing gray matter volume change from visit 1 to visit 2 were created by voxel-wise subtracting the normalized images at visit 1 from the images at visit 2, yielding a measure of increase in atrophy between the two visits. Finally, the gray matter volume maps for visit 1 as well as the gray matter volume change maps (difference in gray matter from visit 1 to visit 2) were smoothed with a 6 mm^3^ × 6 mm^3^ × 6 mm^3^ full-width at half-maximum isotropic Gaussian kernel and used for the following analyses.

Additional to the VBM analyses, the lesion load of corticospinal tract was determined based on FLAIR-weighted MRI images and using the technique outlined in (Klein et al. (2015); Fraunhofer MEVIS NeuroQLab3.531 software package). Changes in the lesion load were assessed by subtracting the lesion load at visit 1 from the lesion load at visit 2.

### Associations Between Gray Matter Volume and Depressive Symptoms

Associations between gray matter volume and depressive symptoms were assessed in four different ways. In all of these four analyses, the magnitude of depressive symptoms (BDI score) or its change between the two visits was voxel-wise correlated with the gray matter volume or its change between the two visits using the “Multiple Regression”-design implemented in SPM8.

First, the cross-sectional relationship between gray matter volume and severity of depressive symptoms was calculated with a correlation analysis between the subjects’ BDI score at visit 1 and the gray matter volume at visit 1.

Second, the relationship between baseline depressive symptoms and prospective development of gray matter volume was calculated with a correlation analysis between subjects’ BDI score at visit 1 and the change in gray matter volume from visit 1 to visit 2 (using the gray matter volume change maps). This calculation tests, if stronger depressive symptoms at visit 1 are associated with future gray matter volume losses in certain brain regions (e.g., due to inflammatory processes).

Third, the relationship between baseline gray matter volume and prospective development of depressive symptoms was calculated with a correlation analysis between subjects’ gray matter volume at visit 1 and the change in BDI scores from visit 1 to visit 2. This calculation tests, if gray matter volume reductions in a certain area at visit 1 are associated with a worsening of depressive symptoms from visit 1 to visit 2.

Fourth, the relationship between gray matter volume *changes* and prospective development of depressive symptoms was calculated with a correlation analysis between subjects’ change in gray matter volume from visit 1 to visit 2 (using the gray matter volume change maps) and change in BDI scores from visit 1 to visit 2. This analysis tests, if the development of gray matter volume in a certain area from visit 1 to visit 2 is related to the development of depressive symptoms from visit 1 to visit 2.

### Associations Between Lesion Load and Depressive Symptoms

Associations between lesion load of the corticospinal tract and depressive symptoms were assessed in the same way as described above for the VBM analyses. Accordingly, we computed correlations between lesion load at visit 1 and BDI scores at visit 1 (cross-sectional relationships), between BDI scores at visit 1 and change in lesion load from visit 1 to visit 2 (prediction of lesion development by depressive symptoms), between lesion load at visit 1 and change in BDI scores (prediction of depression development by baseline lesion load) and between changes in lesion load from visit 1 to visit 2 and changes in BDI scores from visit 1 to visit 2 (“parallel” development of lesion load and depressive symptoms).

### Control Over Potential Confounders and Significance Threshold

Since structural brain volumina are strongly age-dependent, we included age as an additional covariate of no interest for every analysis. Because the development of depressive symptoms might furthermore be dependent on the baseline severity of depressive symptoms, the last two analyses testing the relationship between gray matter volume and prospective development of depressive symptoms were calculated with both, age and BDI scores at visit 1, as covariates. To this end, these variables were included as covariates in the multiple regression design of SPM8.

Because assessments of depression in MS patients may be confounded by depression-like somatic symptoms such as MS-related fatigue, we repeated all analyses using the affective symptoms subscale of the BDI (Steer et al., 1999) instead of the BDI total score. This validation analysis was aimed at minimizing the impact of somatic depressive symptoms on the statistical results. However, in none of the significant areas, results differed substantially between the analyses with the BDI total score and BDI affective symptoms subscale, so that for reasons of simplicity only the results based on the BDI total score are reported in the following.

All analyses were whole-brain analyses without* a priori* regions of interest. Similar to previous VBM studies on depression (Egger et al., 2008; Li et al., 2010; Peng et al., 2011; Liu et al., 2014), the results were considered significant at a significance level of *p* < 0.001 uncorrected with an additional cluster extent threshold of 50 contiguous voxels. Labeling of the significant clusters was carried out using the established advanced anatomic labeling atlas (AAL) and SPM8.

## Results

### Patients: Age, Depressive Symptoms, Medication and EDSS

Initially, 15 male and 29 female MS patients with a mean age of 47.6 years, mean EDSS score of 3.7 and mean BDI score of 10.4 were included. RRMS and secondary-progressive multiple sclerosis (SPMS) patients did not differ significantly in BDI scores at visit 1 (two-sample *t*-Test *T* = 1.35; *p* = 0.89), visit 2 (two-sample *t*-Test *T* = 1.24; *p* = 0.22) or change in BDI scores from visit 1 to visit 2 (two-sample *t*-Test *T* = 1.16; *p* = 0.25).

In the 40 patients with reassessment at visit 2 (four drop-outs), EDSS scores did not change significantly between visit 1 and visit 2, whereas BDI scores decreased by 2.9 points on average (paired *t*-Test *T* = 3.25; *p* = 0.02). BDI and EDSS scores showed a significant cross-sectional correlation at visit 1 (*r* = 0.32, *p* = 0.04), indicating worse depressive symptoms with growing disability. The *change* in BDI scores however was not associated with EDSS scores at visit 1 (*r* < 0.01, *p* = 0.99) or the change in EDSS scores from visit 1 to visit 2 (*r* = 0.04, *p* = 0.82). Moreover, there was no association between the number of relapses and the change in BDI scores from visit 1 to visit 2 (Kendall-Tau rank correlation Tau = −0.30, *p* = 0.82).

The characteristics and medications of the cross-sectional sample (*n* = 44, used for the first analysis) and the longitudinal sample (*n* = 40 due to the four drop-outs, used for the three prospective analyses) are summarized separately in Table 1.

**Table 1:** **Characteristics of patients included in the cross-sectional and longitudinal analyses**.

	Cross-sectional sample Absolute numbers	Longitudinal sample (visit 1) Absolute numbers	Longitudinal sample (visit 2) Absolute numbers
*n*	44	40	40
Sex (male/female)	15/29	15/25	15/25
Antidepressant subscription (yes/no)	5/39	5/35	5/35
Form of MS (RR-MS/SP-MS)	33/11	29/11	29/11
Basic medication	Interferon-beta (10),	Interferon-beta (7),	Interferon-beta (7),
	glatiramer acetate (7),	glatiramer acetate (7),	glatiramer acetate (5),
	natalizumab (7),	natalizumab (7),	natalizumab (5),
	fingolimod (1),	fingolimod (1),	dimethyl fumarate (5),
	teriflunomid (1),	teriflunomid (1),	fingolimod (1),
	azathioprine (1), none (17)	azathioprine (1), none (16)	teriflunomid (1), none (16)
Relapses between visit 1 and visit 2	–	–	None (27), one (8), two (5)
	**Median (Interquartile Range)**	**Median (Interquartile Range)**	**Median (Interquartile Range)**
Age (in years)	49 (16)	49 (18)	50.5 (18)
BDI	8.5 (10)	8 (9)	6 (7)
EDSS	3.75 (2.88)	3.75 (2.88)	3.5 (3)

*BDI, Beck Depression Inventory; EDSS, Expanded Disability Status Scale; RR-MS, Relapsing-remitting multiple sclerosis; SP-MS, secondary-progressive multiple sclerosis*.

### Association Between Baseline Gray Matter Volume and Baseline Severity of Depressive Symptoms

**Figure 1:**
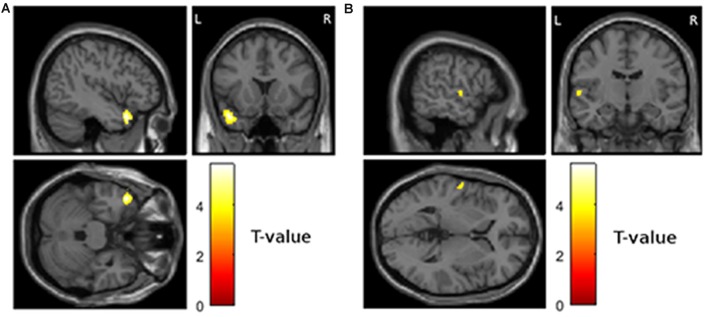
**Areas showing a cross-sectional relationship between atrophy and magnitude of depressive symptoms at visit 1 (*p* < 0.001 uncorrected and more than 50 contiguous voxels in a cluster). (A)** Left temporal pole, MNI coordinates of peak voxel −44, 16, 28 **(B)** left superior temporal gyrus, MNI coordinates of peak voxel −58, −10, 4.

First, we tested if reduced gray matter volume at visit 1 was associated with the magnitude of depressive symptoms at visit 1. This analysis revealed two significant clusters in the left temporal lobe (Figure 1; first cluster MNI peak voxel [*x*, *y*, *z*] −44, 16, −28; *T*-value peak voxel 5.58; cluster extent 508 voxels, AAL labels middle and superior left temporal pole, second cluster MNI = −58, −10, 4; *T* = 4.04; extent = 56; AAL = left superior temporal gyrus).

### Association Between Baseline Depressive Symptoms and Subsequent Development of Gray Matter Volume

Second, we tested if depressive symptoms at visit 1 were associated with gray matter volume losses from visit 1 to visit 2. This analysis revealed no significant results.

### Association Between Baseline Gray Matter Volume and Subsequent Development of Depressive Symptoms

**Figure 2:**
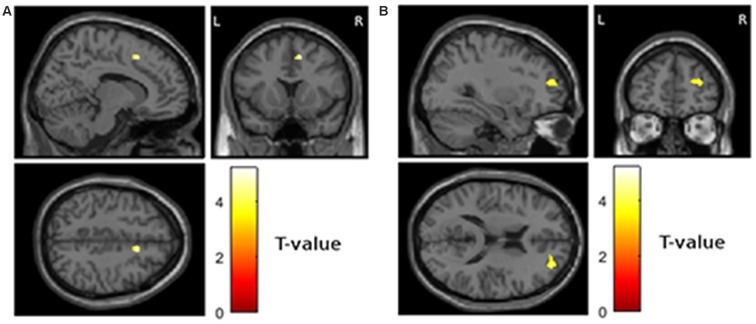
**Areas showing a relationship between atrophy at visit 1 and worsening of depressive symptoms from visit 1 to visit 2 (*p* < 0.001 uncorrected and more than 50 contiguous voxels in cluster). (A)** Right middle cingulate cortex, MNI coordinates of peak voxel 12, 14, 46 **(B)** right middle frontal gyrus, MNI coordinates of peak voxel 32, 50, 16.

Third, we tested if reduced gray matter volume at visit 1 was associated with the worsening of depressive symptoms from visit 1 to visit 2. This analysis revealed significant results for the right cingulate cortex (Figure 2A; MNI = 12, 14, 46; *T* = 5.18; extent = 66; AAL = right middle cingulate cortex) and the right frontal lobe (Figure 2B; MNI = 32, 50, 16; *T* = 3.69; extent = 140; AAL = right middle frontal gyrus).

### Association Between Gray Matter Volume Changes and Development of Depressive Symptoms

**Figure 3:**
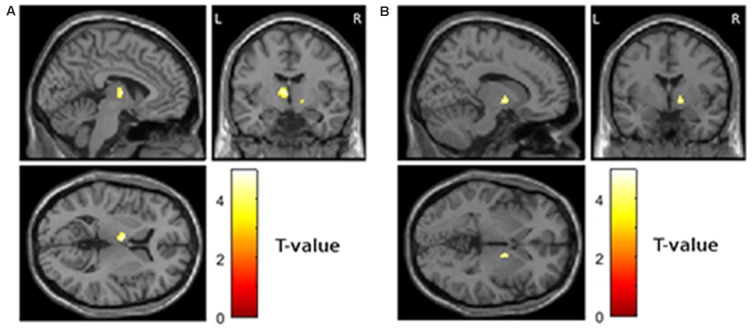
**Areas showing a relationship between increase in atrophy from visit 1 to visit 2 and worsening of depressive symptoms from visit 1 to visit 2 (*p* < 0.001 uncorrected and more than 50 contiguous voxels in a cluster). (A)** Left thalamus, MNI coordinates of peak voxel −5, −8, 6 **(B)** right globus pallidus, MNI coordinates of peak voxel 14, −2, −2.

Fourth, we assessed the relationship between *increase* in atrophy and worsening of depressive symptoms over time. This analysis revealed significant clusters in the left mediodorsal nucleus of the thalamus (Figure 3A; MNI −5, −8, 6; *T* = 5.16; extent = 175; AAL = left thalamus) and the right internal pallidum (Figure 3B; MNI 14, −2, −2, *T* = 4.35; extent = 90; AAL = right globus pallidus).

### Associations Between Lesion Load and Depressive Symptoms

Similar to our analyses of VBM data, we tested cross-sectional and prospective relationships between the lesion load of the corticospinal tract and depressive symptoms. Here, no significant cross-sectional or longitudinal dependencies were found.

## Discussion

In this prospective study, we tested cross-sectional and prospective associations between brain gray matter volume and depressive symptoms in a sample of MS patients. The specific design of our study, including MRI scans at visit 1 and visit 2, allowed for the first time a combined analysis of distinct temporal aspects of the relation between depression and brain atrophy: we could distinguish “dynamical” aspects, e.g., a concomitant development of depression and brain atrophy, from “predictive” aspects (how the future course of depression is predicted by certain patterns of atrophy) and “static” aspects (e.g., cross-sectional relationships at one point in time).

We found a remarkable association between growing atrophy in the left thalamus and the right internal globus pallidus and worsening of depressive symptoms over time. Reduced gray matter volumes of the right middle cingulate cortex and middle frontal gyrus predicted a future worsening of depressive symptoms. Furthermore, we could replicate previously reported cross-sectional (static) relationships between regional brain atrophy in the left temporal lobe and stronger depressive symptoms.

The results fit well into the concept of a disturbed cortico–striatal–pallido–thalamic loop in depression (Price and Drevets, 2012). Based on studies on depression-related brain atrophy, this concept has also been employed on depression in the context of neurological diseases, particularly MS: “What may therefore link depression in a disparate set of neurologic disorders is a disruption in neural circuits originating in the prefrontal cortex and then sequentially relaying in the striatum, globus pallidus, and thalamus, before looping back to its prefrontal site of origin” (Feinstein et al., 2004). Consistently, the two areas whose growing atrophy was associated with growing severity of depressive symptoms, (dynamic relationship in our study), i.e., the internal globus pallidus and the mediodorsal thalamus, are closely connected and part of a limbic/motivational basal ganglia circuit (Nieuwenhuys et al., 2007). Central to our results, it has been reported, that lesions in the globus pallidus potentially lead to anhedonia and depression (Vataja et al., 2001; Miller et al., 2006; Vijayaraghavan et al., 2008; Adam et al., 2013), whereas deep brain stimulation of the pallidum can alleviate these depressive symptoms (Kosel et al., 2007 for a case report, Vidailhet et al., 2005, for non-significant trend in 22 patients). Both findings are consistent with a suggested involvement of the globus pallidus in a dopaminergic reward circuit (Hong and Hikosaka, 2008) that in turn constitutes a convenient framework for the understanding of the reported relationship between progressive atrophy of this region and the course of depressive symptoms. In such a framework, the reward-representing capacities of the globus pallidus might diminish by its progressive atrophy, thereby possibly promoting apathy and anhedonia as central depressive symptoms. A potentially clinical relevant notion is that this anhedonia associated with pallidum lesions can partly be reversed by dopaminergic modulation (Adam et al., 2013).

A reduced gray matter volume in the right prefrontal cortex predicted a future worsening (or missing improvement) of depressive symptoms. This finding is consistent with previous work by Gobbi et al. (2014), who also documented atrophy in the right dorsolateral and inferior frontal cortex in MS patients with depressive mood using VBM. A (speculative) explanation for the relationship between prefrontal atrophy and depression proneness might be explained by a diminished capacity to adapt to and cope with (disease-related) restrictions in subjects with more pronounced prefrontal atrophy. This also ties with the finding that depression in MS-patients is associated with impaired executive functions (Feinstein, 2006) that are in turn dependent on the integrity of the prefrontal cortex (Morgen et al., 2006).

In our study, we found a cross-sectional (static) correlation between magnitude of depressive symptoms and left anterior temporal lobe atrophy. The previous cross-sectional research yielded similar results on atrophy in the temporal pole, but sometimes also in the frontal lobe and partly in the right and not the left hemisphere (Feinstein et al., 2014). A recent study using cortical thickness measures documented atrophy in depressed MS patients both in the temporal and the frontal cortex bilaterally (Nygaard et al., 2015). Hence, it seems reasonable to argue that the anterior temporal lobe and, probably, frontal areas play a role in MS-related depression, but results are yet too inconsistent to argue for a functional difference between both brain hemispheres in this regard.

Our study was carried out on a sample of MS patients, in which both depression and gray matter loss occur more frequent than in the general population. Nevertheless, the patterns of depression-related atrophy in our study closely resemble that reported for depressive patients without somatic disease (Du et al., 2012, for a meta-analysis), thereby suggesting similar relationships between gray matter changes and depressive mood in subjects with and without underlying MS.

Interestingly, average depressive symptoms did not worsen over time but rather improved in our sample of MS patients. This finding is in line with a recent longitudinal study on the development of depression in MS, reporting stable or improving depressive symptoms in the long term (Koch et al., 2015).

The primary clinical measure of our study were changes in subclinical depression, which is very common in MS patients and thus a highly interesting and yet under investigated topic. However, one should bear in mind that we did not investigate cases of “full”, clinically diagnosed depression, but related symptom scores to gray matter densities in a correlational approach. Thus, the validity of the results for clinical cases is uncertain.

Taken together, our results suggest increasing damage of subcortical motivational systems as a risk factor for the development of depression in MS patients as well as (intact) prefrontal function as a potential resilience factor. Prospective studies are needed aiming at predicting the development of depressive symptoms on basis of impaired prefrontal functions and at preventing worsening of depressive mood. Here, an application of dopaminergic antidepressants like for example bupropion might be a promising approach considering the depression-related atrophy in dopaminergic motivational systems, whereas behavioral therapy focusing on executive deficits might help to counteract prefrontal impairments.

## Author Contributions

KH, AK, HH, JK, JH and HS: design of the study. KH, FW and HH : data collection. HS, HH, JK and FW: statistical analyses. HS and HH: writing of the article. HS, KH, JH, JK, FW, AK and HH: article correction and final approval.

## Conflict of Interest Statement

The authors declare that the research was conducted in the absence of any commercial or financial relationships that could be construed as a potential conflict of interest. The handling Editor declared a shared affiliation, though no other collaboration, with several of the authors HH, KH, FW and states that the process nevertheless met the standards of a fair and objective review.
